# Unusual Response of Subclavian In-Stent Restenosis to Balloon Angioplasty in a Patient with HIV

**DOI:** 10.1155/2015/157623

**Published:** 2015-01-18

**Authors:** Mohammad Atif Rana, Jagan Beedupalli, Nuri I. Akkus

**Affiliations:** Louisiana State University, 1501 King Highway, Shreveport, LA 71130, USA

## Abstract

Human Immunodeficiency Virus (HIV) infection and use of protease inhibitors have been associated with accelerated atherosclerosis. Increased rates of coronary in-stent restenosis are reported in these patients. There is limited data available on peripheral vascular disease interventions on these patients. Herein we report an aggressive subclavian in-stent restenosis with an unexpected response to balloon angioplasty treatment with a large, mobile tissue flap formation and its treatment with another stent.

## 1. Introduction

The survival of patients with Human Immunodeficiency Virus (HIV) infection has dramatically increased with advent of highly active antiretroviral therapy (HAART). Serious metabolic problems with the use of antiretroviral drugs in addition to the HIV infection itself might be causative in accelerated atherosclerosis and unexpectedly higher rates of coronary in-stent restenosis rates in these patients [1, 2]. Although there have been several reports of accelerated coronary atherosclerosis causing unexpected higher restenosis rates after percutaneous coronary interventions in HIV patients [1–5], there has been no report of accelerated restenosis or types of restenosis in peripheral vasculature.

Herein, we report an HIV infected patient with an occluded symptomatic left subclavian artery which had been successfully treated with stenting who developed subsequent aggressive in-stent restenosis that was resistant to balloon angioplasty (BA) and responded to BA with the tear of the neointimal tissue causing a large mobile tissue flap requiring repeat stenting to prevent embolic complications.

## 2. Case Report

**Figure 1:**
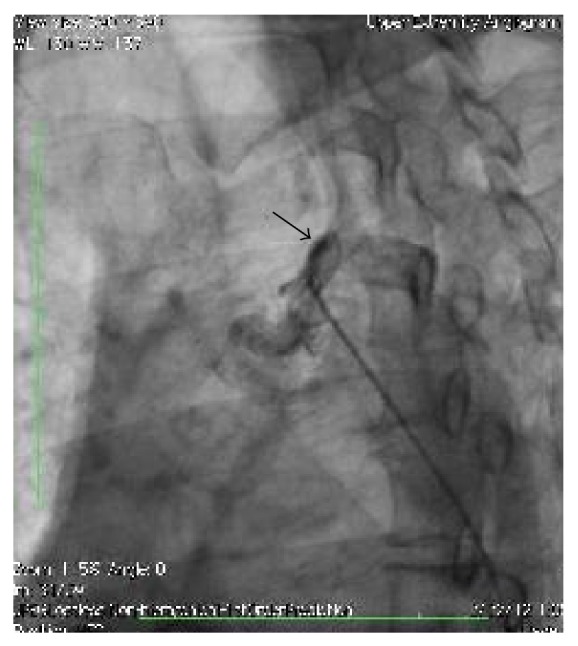
Angiogram showing occluded proximal left subclavian artery.

**Figure 2:**
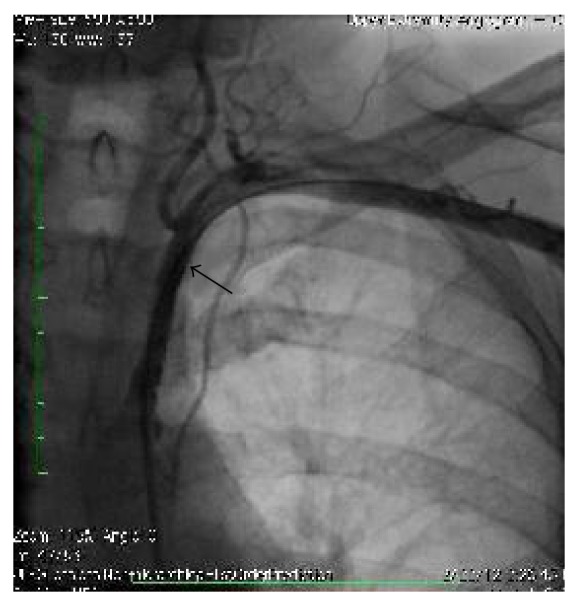
Angiogram after stent placement.

A 56-year-old Caucasian male with hypertension, hyperlipidemia with LDL of 78 on day of procedure, HIV infection being currently treated with HAART (Ritonavir 100 mg a day, emtricitabine/tenofovir disoproxil 200/300 mg a day, and darunavir 400 mg a day) with a CD4 count of 420, coronary artery disease status after implantation of a drug eluting stent in an obtuse marginal two years ago, asymptomatic bilateral carotid artery stenosis, left subclavian artery occlusion with subclavian steal status after percutaneous angioplasty, and stenting with a Visi-Pro 7.0 × 37 mm stent (Covidien) one year ago (Figures 1 and 2) presented with recurrent complaint of left upper extremity claudication and occasional lightheadedness on using left arm for the last 6 months. He continues to smoke one pack of cigarettes per day and denies any alcohol or illicit drug abuse. He was compliant with his medications which also included aspirin 325 mg daily, plavix 75 mg daily, rosuvastatin 20 mg daily, lisinopril 20 mg daily, and famciclovir 500 mg daily in addition to HARRT. Physical exam was significant for a blood pressure of 111/76 mm Hg on his right arm and 82/50 mm Hg on his left arm with diminished pulses in his left upper extremity compared with his right upper extremity.

A carotid Doppler study done 3 months ago to evaluate his carotid arteries revealed evidence of left subclavian steal phenomenon along with 50–69% stenosis of right internal carotid artery and >70% stenosis of the left internal carotid artery.

**Figure 3:**
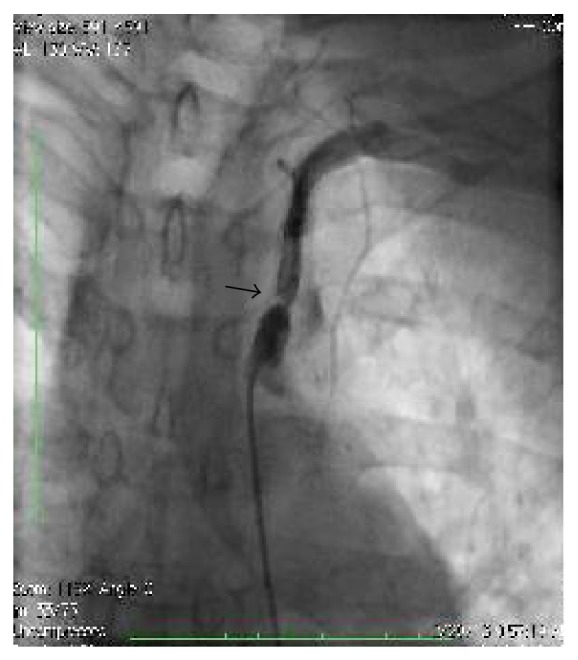
Angiogram showing significant in-stent stenosis.

With this clinical picture, he underwent a left subclavian angiography that revealed a 95% eccentric in-stent restenosis of the proximal portion of left subclavian stent (Figure 3). We then proceeded with intervention of left subclavian in-stent restenosis.

**Figure 4:**
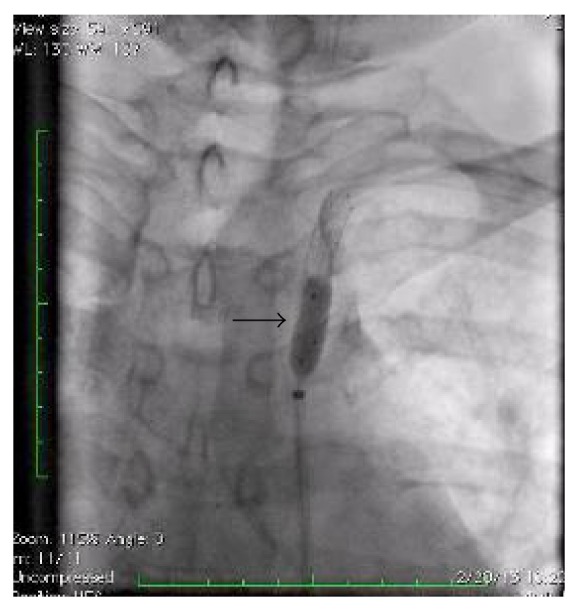
Angiogram showing balloon angioplasty.

**Figure 5:**
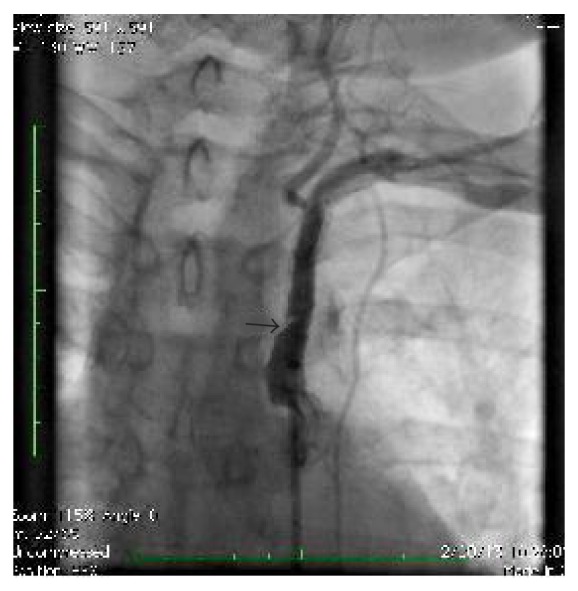
Angiogram showing mobile fractured in-stent stenosis in systole.

**Figure 6:**
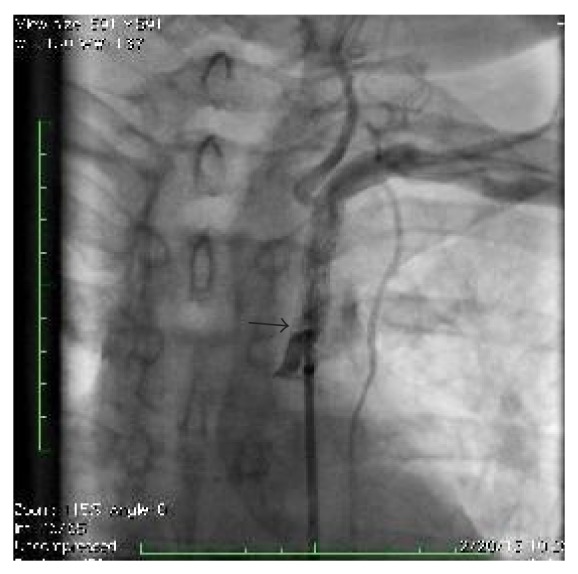
Angiogram showing mobile fractured in-stent stenosis in diastole.

**Figure 7:**
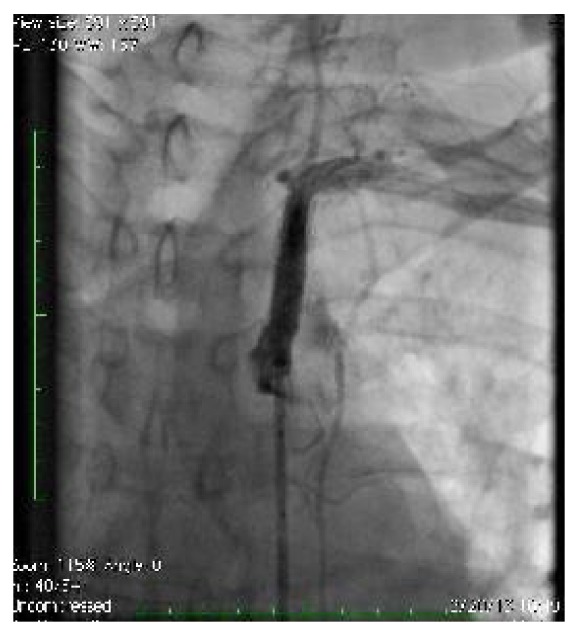
Angiogram after stent placement showing trapped in-stent restenosis.

The left subclavian artery was engaged with a 6 French 80 cm Shuttle sheath and after therapeutic anticoagulation achieved with heparin, the in-stent restenosis lesion was crossed with a Prowater 300 cm wire (Abbott Vascular, IL, USA) and angioplasty of the lesion was performed with a Viatrac 7.0 × 15 mm balloon (Abbott Vascular, IL, USA) inflated at 14 atmospheres pressure (Figure 4). After balloon angioplasty, a tear in the extensive neointimal tissue creating a highly mobile tissue flap was noted (Figures 5 and 6). To prevent distal embolization, it was covered with Express SD 7.0 × 15 mm stent (Boston Scientific Corporation) deployed at 12 atmospheres (Figure 7). Then, the overlap area with previous stent was dilated with same stent balloon inflated at 14 atmospheres. After stenting, satisfactory results were obtained with no complications. Blood pressure was equalized after the intervention with right arm blood pressure of 120/80 and left arm blood pressure was 118/80. The patient also had coronary angiogram at the same time to evaluate coronary stent placed in obtuse marginal 2 years ago and it was patent without any in-stent restenosis. After the procedure, patient was discharged home with recommendations to continue on dual antiplatelet therapy for at least one month and to institute risk factor modification including smoking cessation. Patient was asymptomatic at 3 months of follow-up.

## 3. Discussion

In-stent restenosis is an increasingly recognized problem in patients with HIV infection. It is caused by excessive smooth muscle proliferation and accumulation of extracellular matrix [1]. Increased repeat coronary revascularizations for severe, diffuse in-stent restenosis are reported in HIV patients [1], which suggests accelerated and premature atherosclerosis in these individuals. It is not clear whether smooth muscle cell proliferation and the accumulation of extracellular matrix, which are the main processes involved in in-stent restenosis, may be induced by protease inhibitors or by the HIV itself [1, 4].

Studies have shown that patients with higher levels of inflammatory markers, such as C-reactive protein, at the time of PCI have higher restenosis rates [3, 4]. HIV infected patients have high levels of C-reactive protein compared to age and sex matched controls [3]. Chronic low-level inflammation in HIV patients may therefore contribute to their high rate of restenosis [3]. By inducing adhesion molecule expression on endothelial cells and LDL cholesterol uptake by macrophages, C-reactive protein may contribute directly to atherogenesis and restenosis [3, 4].

Further, HIV, in itself, is an atherogenic state. HIV disease induces accelerated T-cell proliferation, heightened T-cell activation, and high levels of inflammatory marker expression and immunologic perturbations that persist even after the introduction of antiretroviral therapy. T lymphocytes and inflammatory cytokines play key roles in atherosclerosis. Thus, immunodeficiency and immune reconstitution may accelerate atherosclerosis and in-stent restenosis [3]. Necropsy studies had demonstrated premature CAD in HIV-infected patients even before the advent of protease inhibitors postulating that both HIV infections by itself and HAART may be causative.

On the other hand, though highly active antiretroviral therapy (HAART) treatment has led to a dramatic decline of morbidity and mortality in HIV infected patients, it is also associated with significant metabolic adverse effects, that is, alteration of lipid profile, insulin resistance, and impaired endothelial function [6–8]. Alteration of lipid metabolism leads to reduced uptake of serum lipids by adipocytes, increased lipolysis in the subcutaneous adipose tissue, and increased production of lipids by hepatocytes [5]. In addition, protease inhibitors such as ritonavir, indinavir, and amprenavir upregulate CD36, a scavenger receptor that mediates cholesterol uptake in macrophages causing aggressive atherosclerosis and in-stent restenosis [5]. Although HIV infection causes immunosuppression with attenuated inflammatory response to certain opportunistic infections, HIV infection causes profound functional alterations of the endothelium, resembling the subclinical inflammation in atherosclerosis. Interestingly, animal studies have shown concomitant thickening of neointima and thinning of medial tissue leading to increased neointima/media ratio. Previous studies have shown contribution of altered apoptosis as a consequence of HAART therapy as suggested by increased transcript levels of the proapoptotic BH3-only protein Noxa, which has been shown to be regulated at the transcriptional level in injured vessels of HAART treated animals [9, 10]. Also, leukocyte adherence to endothelium is enhanced as the expression of cell adhesion molecules increases [2].

## 4. Conclusion

All these above-mentioned mechanisms contribute to aggressive form of in-stent restenosis in HIV infected patients. In contrast to coronary arteries, the peripheral vasculature can harbor excessive amounts of smooth muscle proliferation and extracellular matrix at sites of in-stent restenosis. Conventionally, balloon angioplasty has been the treatment of choice for in-stent restenosis in peripheral vasculature. But, in this particular set of patients, balloon angioplasty may not always be adequate and can lead to complications like dissection of neointimal hyperplasia within in-stent restenosis that may require stenting to prevent distal embolization as illustrated in our case.

Aggressive risk factor modification and lipid-lowering drug therapy including statins are required in this subset of patients to prevent future cardiovascular events. Future directions in treating these patients need to be explored. One possibility would be the use of drug eluting stents rather than bare metal stents in peripheral vasculature in these patients that would reduce the risk of aggressive in-stent restenosis as observed in this patient. This was reinforced by lack of any in-stent restenosis observed in drug eluting stent placed in obtuse marginal coronary artery 2 years ago in our patient.
